# Behavioural optimisation to address trial conduct challenges: case study in the UK-REBOA trial

**DOI:** 10.1186/s13063-022-06341-6

**Published:** 2022-05-12

**Authors:** Louisa Lawrie, Eilidh M. Duncan, Jan O. Jansen, Marion K. Campbell, Evelyn Brunsdon, Zoë Skea, Taylor Coffey, Claire Cochran, Katie Gillies

**Affiliations:** 1 Institute of Applied Health Sciences, School of Medicine, Medical Sciences and Nutrition, Health Services Research Unit, 3Rd Floor Health Sciences Building, University of Aberdeen, Foresterhill, Aberdeen, AB25 2ZD UK; 2 Center for Injury Science, University of Alabama at Birmingham, Birmingham, AL 35294 USA

**Keywords:** Trial methodology, Behavioural science, Complex interventions, Process evaluations, Theoretical domains framework

## Abstract

**Background:**

Clinical trials comprise multiple processes at various stages of the trial lifecycle. These processes often involve complex behaviours such as recruiting vulnerable patient populations and clinicians having to deliver complex trial interventions successfully. Few studies have utilised a behavioural framework to assess challenges and develop strategies for effective trial recruitment and delivery of trial interventions. This study reports the application of an innovative methodological approach to understand core trial processes, namely recruitment and intervention delivery, using a behavioural science approach to develop strategies designed to mitigate trial process problems.

**Methods:**

The UK-REBOA trial aims to evaluate the clinical and cost-effectiveness of resuscitative endovascular balloon occlusion of the aorta (a novel intervention) in injured patients with exsanguinating haemorrhage. A behavioural investigation (‘diagnosis’) was conducted using theory-informed (Theoretical Domains Framework, TDF) semi-structured interviews with site staff from the UK-REBOA trial to examine trial processes which could be improved in relation to trial recruitment and delivery of the intervention. Interviews were analysed using the TDF to identify influences on behaviour, which were then mapped to techniques for behaviour change and developed into potential solutions.

**Results:**

The behavioural diagnosis of the challenges experienced during trial processes highlighted factors relevant to a range of TDF domains: Skills, Environmental context and resources, Beliefs about capabilities, Beliefs about consequences, Social influences, and Memory, attention, and decision-making processes. Within the solution development phase, we identified 24 suitable behaviour change techniques that were developed into proposed solutions to target reported process problems with the aim of changing behaviour to improve recruitment and/or intervention delivery. Proposed solutions included targeted changes to trial training content, suggestions to restructure the environment (e.g. reinforced the purpose of the trial with information about the social and environmental consequences) and other strategies to reduce barriers to recruitment and intervention delivery.

**Conclusions:**

This study demonstrates the feasibility of applying a behavioural approach to investigate (‘diagnose’) behavioural trial process problems and subsequently develop and implement targeted solutions (‘treatment’) in an active trauma trial. Understanding the factors that affected behaviour, attitudes and beliefs in this trauma trial allowed us to implement theoretically informed, evidence-based solutions designed to enhance trial practices.

**Trial registration:**

ISRCTN 16,184,981

**Supplementary Information:**

The online version contains supplementary material available at 10.1186/s13063-022-06341-6.

## Introduction

Randomised controlled trials are the best method of evaluating the effectiveness of interventions and remain a cornerstone of evidence-based healthcare. The successful delivery of randomised controlled trials relies on the enactment and progression of multiple, often connected, processes across several stages of the trial lifecycle. Trial processes include, but are not limited to, research question conception, trial design, recruitment, intervention delivery, data collection, retention of study participants, analysis, dissemination of findings, and close down. Many of these processes have been identified (and remain) as methodological priorities for the trial community [1, 2]. Trial processes often involve various people (e.g. patients, clinicians, trial managers) performing an action (e.g. approaching eligible patients, delivery of the trial intervention or returning a questionnaire). These actions need to be performed effectively for a trial to be delivered successfully, but the literature shows that trials often fail to deliver on many of these components. These trial behaviours can be complex, often determined by the specific context, but importantly are often largely amenable to change. Identifying the behavioural influences of core trial processes (and optimising them where improvements are needed) could help contribute to understanding the overall success or failure of the trial. Behavioural approaches to understand and change trial process behaviours are starting to emerge in the literature, but to date have largely focussed on identifying behavioural problems for trial recruitment and retention [3–6]. The applicability of a behavioural science approach to both identify trial process barriers *and* implement strategies to address these challenges warrants further attention.

In order to develop the methodology around using behavioural science to explore problems of trial processes, we applied a behavioural framework to inform the process evaluation for a pragmatic effectiveness complex intervention trial, the UK-REBOA trial. The UK-REBOA trial compares the effectiveness of resuscitative endovascular balloon occlusion of the aorta (REBOA) in addition to standard major trauma centre care for patients with suspected life-threatening torso haemorrhage within the NHS. Trauma trials (and also clinical care in trauma) rely on individuals functioning within large trauma teams in complex, fast-moving clinical environments that require rapid decision-making in order to avoid errors that could be life-threatening [7]. As such, trial delivery in this setting relies on the cumulative, multi-component, often simultaneous, behaviours both within and across people. For example, trauma team leaders (TTL) have to make rapid judgements (often in minutes) on multiple eligibility criteria to ensure trial recruitment in a timely manner. At the same time, other trial staff assist TTLs in recruitment by performing multiple behaviours, such as flagging a ‘code red’ patient (requiring activation of the Massive Transfusion Protocol) and ensuring the appropriate equipment is ready for potential randomisation and intervention delivery. The complexities of the clinical context may make the already challenging problem of recruitment or delivery of an unfamiliar intervention more problematic in this emergency care setting. Whilst recent studies have explored the challenges to recruitment in emergency trauma trials, they have not been considered within a behavioural theory or framework, nor have they developed and implemented evidence-based solutions to potentially address the challenges identified [8, 9].

By investigating trial process barriers and enablers through a behavioural lens, we could assess who needs to do what differently, to whom, when and how, as well as apply these theory-informed findings to develop evidence-based solutions based on behaviour change science. Recent studies have highlighted the utility of the Theoretical Domains Framework (TDF) to identify behavioural processes where the performance could be improved within clinical trials; we therefore chose to apply this framework in our study [3–5, 10, 11]. Application of the TDF also provides an opportunity to examine the behaviours which need to change in order to improve the conduct of a trial and represents the first step in the process of developing behaviour change interventions [12]. Thus, using the TDF to ‘diagnose’ and subsequently ‘treat’ challenges within a trial could help to improve the effectiveness and delivery of trial processes. Application of the TDF in a trial context is particularly promising given the evidence which highlights the effectiveness of utilising this framework to understand the origins of healthcare behaviour [13–15]. Potential solutions to overcome barriers identified using the TDF can be developed by incorporating evidence-based Behaviour Change Techniques (BCT) to improve the delivery of trial processes as they have been for other behaviours related to healthcare [15, 16].

The current study aimed to conduct a behavioural analysis to identify the trial process problems that impact on successful trial recruitment and intervention delivery within the UK-REBOA trial, and develop and implement behavioural solutions to address the barriers reported.

## Methods

### Context: the UK-REBOA trial

The UK-REBOA trial is evaluating the effectiveness of resuscitative endovascular balloon occlusion of the aorta (REBOA) in addition to standard major trauma centre care for patients with suspected life-threatening torso haemorrhage within the NHS. Suspected life-threatening torso haemorrhage is rare; thus, numbers of eligible patients are small and do not present often. The trial intervention is also complex — it involves the insertion of a REBOA catheter through the femoral artery (cannulation), which can be difficult in severely hypovolemic patients; guiding the catheter to a location thought to be above the site of the bleeding and inflation of the catheter balloon at that location (subsequent passage and inflation of the intravascular balloon occlusion device); management of the balloon in situ (whilst ensuring rapid transfer to theatre); and finally successful deflation and removal of the catheter. This is a complex procedure requiring high technical skill. See Additional file 1 for a visual depiction of the REBOA procedure.

### Study design

The study reported in this manuscript involved a theoretically informed behavioural investigation, using semi-structured one-to-one interviews with clinical site staff across a number of sites. The study comprised two phases: (1) phase 1 interviews were conducted during the pilot stages of the REBOA trial to identify initial difficulties associated with the set-up and initiation of trial processes (including recruitment) across the first active sites; (2) phase 2 interviews were conducted when sites had obtained more experience of the trial, randomising participants, and deploying the intervention. Phase 2 was more specifically designed to identify the behavioural challenges associated with the trial processes of recruitment and intervention delivery and to develop targeted solutions to address said challenges.

### Participants

Interview participants included clinical staff who were involved in the REBOA trial across different UK sites and who occupied various roles (both clinically and for the trial), such as trauma consultants, surgeons, registrars and research nurses. A total of 49 invitations were sent across both interview phases with the aim of recruiting a diverse sample which was informed by five key sampling aspects of information power, which suggests that a focussed aim (as in this study), concentrated specificity of the sample (i.e. those involved in recruitment and intervention delivery), application of established theory, rich narratives provided and no cross-case analysis all supported a smaller sample [17].

### Data collection

Separate topic guides were used for each phase of the study. The topic guide in phase 1 (see Additional file 2) was designed to explore site staff decisions to become involved with the trial, views about the rationale for the trial, the recruitment process at their site and the consent process. The topic guide used in phase 2 (see Additional file 3) covered similar areas but was informed by the TDF and focussed on recruitment and intervention delivery — i.e. deployment and insertion of the REBOA catheter (issues that had been identified as core in phase 1). The TDF is an established behavioural framework that integrates 33 theories of behaviour into 14 domains that inhibit or enable behaviour (Knowledge, Skills, Social/Professional Role and Identity, Beliefs about Capabilities, Beliefs about Consequences, Optimism, Reinforcement, Intentions, Goals, Memory/Attention/Decision-making Processes, Environmental Context and Resources, Social Influences, Emotion, Behavioural Regulation) [13]. Both topic guides were refined by the research team and updated iteratively to ensure robustness.

Recruitment in phase 1 targeted the first six sites to enact randomisation for the REBOA trial. Sites invited to participate in phase 2 were those which had either recruited a number of patients into the trial, experienced notable difficulties with recruitment, had recently randomised a patient to the trial and/or reported a missed opportunity to recruit an eligible patient. Email invites were distributed to potential participants by the trial manager (CC) on behalf of the co-chief investigators (JJ, MC). Two attempts were made to engage eligible site staff. A member of the research team (EB, ZS, LL) then scheduled a mutually convenient time for a telephone or Microsoft Teams interview [18].

Phase 1 interviews were conducted by EB (medical anthropologist) and ZS (health services researcher) between May 2018 and April 2019. Phase 2 interviews were conducted by LL (female, academic researcher/psychologist) in October 2020. All participants were aware that the interviewers were neither clinicians nor involved in the daily conduct of the REBOA trial. Interviews were audio-recorded and transcribed verbatim by an external transcription service. Informed written consent was obtained from all participants.

### Data analysis

#### Identification of salient TDF domains

Data from both phases of the study were subjected to the same analysis processes. NVivo 11 was used to facilitate data analysis [19]. A TDF coding guide was used to aid data interpretation: this was developed and iteratively updated during the coding process (LL, ED (health psychologist), KG (trial methodologist)). One researcher (LL) coded transcribed data into the relevant TDF domains. Three of the 18 interview transcripts were independently double coded (LL and TC) and exhibited a large degree of agreement across the double coding. Any disagreements were resolved by a third researcher (KG).

After coding data into TDF domains, belief statements (representative descriptions of utterances across participants) were generated (LL) [16]. Belief statements were designed to present detail on how each domain may be influencing the behaviours of interest, namely (1) recruitment of patients to the REBOA trial and (2) delivery of the REBOA intervention. The research team (ED, KG, TC, LL) collectively discussed the belief statements to agree they were an accurate representation of the quotes coded within each domain.

We used existing TDF analysis methods to identify the domains that were most likely to influence the target behaviours [16]: this included (1) the frequency of belief statements across all domains (statements with a frequency of > 75% were considered most ‘relevant’ as per other TDF-based studies [5]); (2) evidence of strong beliefs that influence the behaviours (i.e. the strength of conviction illustrated by participants during the interviews); and (3) the presence and prevalence of conflicting beliefs. This resulted in some domains that contained frequently reported belief statements not being identified as salient as there was no evidence of strong beliefs, from interviews, that influenced the target behaviours or conflicting beliefs within the domain. Prior to the identification of potential solutions to mitigate trial challenges, we reviewed the barriers relevant to all domains that were amenable to change within the scope of this project. We omitted those that required wider infrastructure changes (e.g. such as a lack of additional personnel to support recruitment) or were not amenable to change (i.e. low number of eligible patients). All criteria were evaluated concurrently (via group consensus) to judge the relevance of each domain.

#### Identification of Behaviour Change Techniques and development of potential solutions to help improve trial processes

Following the identification of the salient domains, components of potential solutions were determined using a standardised process that involved mapping the relevant theoretical domains to Behaviour Change Techniques (BCTs) using the Theory and Techniques Tool [20, 21]. BCTs are defined as the smallest active ingredient of a behavioural intervention (referred to as solutions) such as incentives or goal setting [22]. The BCTs identified as potentially relevant for selected TDF domains were collated, discussed by the research team and adapted to the clinical context of the UK-REBOA trial (LL, ED, KG, TC). In addition, existing training and support materials provided to REBOA site staff were reviewed (LL, KG, ED) to examine the presence of BCTs that may already be delivered in the trial as an opportunity to enhance relevant existing BCTs delivered.

BCTs proposed by the research team were presented at a meeting with the trial manager and chief investigators to discuss the applicability of selected BCTs to support specific trial behaviours (recruitment and intervention delivery). We applied the APEASE criteria (Acceptability, Practicability, Effectiveness, Affordability, Side-effects, and Equity) to support the final selection of the content and mode of delivery for the potential solutions to improve the trial processes [12].

During solution development, training materials were updated in response to the findings of the behavioural investigation and implemented in follow-on training for sites. Training delivery with regard to BCT content was assessed by observation with feedback provided to the training team post-session (KG). Attendees were also asked in their feedback to consider the main message they had taken away from the training in order to determine the most salient aspects of the training content and whether updated content was being received as intended.

**Fig. 1:**
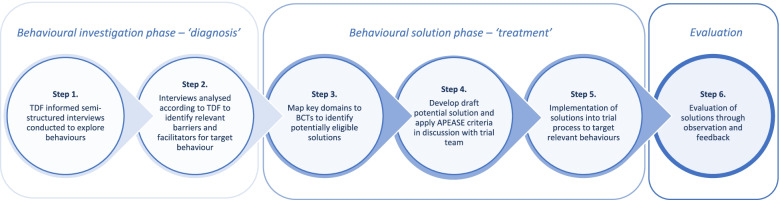
Stages involved in the diagnosis and treatment of issues in REBOA recruitment and intervention delivery. TDF Theoretical Domains Framework, BCT Behaviour Change Technique, APEASE Affordability, Practicability, Effectiveness/cost-effectiveness, Acceptability, Side-effects/safety, Equity

An illustrative diagram which details the key steps involved in this study is summarised in Fig. 1.

## Results

### Sample characteristics

Seventeen participants were interviewed across both phases which included participants from 8 sites and the majority identified as trauma consultants (*n* = 7, 41.18%) (see Table 1). One participant was interviewed in both phases 1 and 2 as they provided initial perspectives on early process problems and later experiences of more established trial process problems. Taken together, the interviews lasted an average of 37 min, ranging between approximately 22 min and 1 h.

**Table 1:** Participant demographics for both phases of the study

Characteristic	Phase 1	Phase 2	Total
*N*	13	5	18 ^a^
*Sites*	5	4	8 ^a^
*Roles*			
Trauma consultant	6	2	7 ^a^
Trauma surgeon	2	-	2
Trauma registrar	2	-	2
Research nurse	2	1	3
Radiologist	1	-	1
Trauma anaesthetist	-	2	2

^a^1 participant interviewed in both phases 1 and 2

The behavioural diagnosis of trial process problems for recruitment of patients in the UK-REBOA trial and in the delivery of the REBOA intervention is described below. The proposed behavioural solutions, designed to mitigate challenges and enhance opportunities (process problem ‘treatment’), are then presented.

### Behavioural investigation: diagnosing the trial process problems for trial recruitment and intervention delivery

Six of the 14 TDF domains were considered relevant to the processes of recruitment in the UK-REBOA trial and to the processes entailed in delivering the trial intervention (the deployment of the REBOA catheter), specifically Skills, Environmental context and resources, Beliefs about capabilities, Beliefs about consequences, Social influences, and Memory, attention, and decision-making processes. Thirty-eight belief statements were identified across the six domains. The TDF domains are presented in detail below. An extended table containing the content and frequency of all TDF domains and associated belief statements is provided in Additional file 4.

### Skills required for successful recruitment and intervention delivery

The skill in recognising a patient who might benefit from REBOA (and thus who would be eligible to be randomised) was reported by participants to influence both recruitment of patients to the trial and delivery of the REBOA intervention.

… you need to have had a reasonable, you know, a good few years of resus [resuscitative] experience to be able to recognise a very sick, bleeding trauma patient and who might benefit from that point of view. Participant 17, trauma consultant, site 6.

However, some participants deemed the ability to recognise eligible patients as less of a barrier to trial recruitment and more of a generic professional skill-set that is common to certain roles within trauma care:

I think you need the generic professional skill of recognising what a critically sick bleeding patient looks like, but that skill I would say is common…it’s common to the skill set of people working on the front line in modern trauma care, so ED [emergency department] positions and trauma anaesthetist. Participant 9, anaesthetist, site 8.

Whilst participants described the process of delivering the REBOA intervention as technical, it was also deemed to be a transferrable skill that may be developed overtime through the delivery of similar interventions. Relatedly, concerns about maintaining competency due to the low frequency of potentially eligible patients who require REBOA was linked to some of the reported issues surrounding the insertion of REBOA and recognising patient eligibility outside of a simulated context:

… but I think ultimately the issue is going to be numbers and maintaining training competencies in a system that less than a third inclusion criteria come much reduced. You know maintaining competence. Participant 10, trauma consultant, site 3.

### Environment, context and resources impacts on recruitment and intervention delivery

In addition to the reported skill-based difficulties in maintaining competency due to low throughput of cases, the scarcity of potential REBOA cases was also referenced as adding a further layer of complexity to recruitment and intervention delivery.

I think another difficulty with this group of patients, is we’re looking at the absolute tip of the iceberg, in terms of the severity of trauma patients, so it’s relatively rare that patients are that sick. It might be 5% of all of them – the code red patients. The code red patients at [hospital], which I think is pretty busy, we’ve got maybe four or five a week. You’re talking about an event that happens maybe once a month, maybe less. Participant 6, clinical research fellow, site 5.

One participant highlighted the contextual differences in patient demographics across various emergency departments in the UK, with some experiencing a greater throughput of potential REBOA cases and the direct influence this has on recruitment potential.

The majority of participants indicated that the ability to both recruit patients to the trial and deliver REBOA depended on staff availability. In terms of recruitment and the intricacies involved in key processes such as screening, many participants highlighted the value of research nurses and clinical fellows, sometimes citing the lack of availability of individuals occupying these specific roles as a barrier. Similarly, the lack of staff available on a 24/7 basis who are trained in the conduct of the trial and delivery of the REBOA intervention was also cited as a barrier to recruitment and intervention delivery.

I think what I really mean is that randomisations of the trial might not be available 24/7 in our hospital because at any one point in the cycle or the clock, you may not have somebody on there that’s trained in the methodology of the trial or the intervention. Participant 9, site 8, trauma anaesthetist.

The clinical context of REBOA (i.e. emergency trauma care) was noted by participants as inherently stressful and fast paced, which could sometimes act as a barrier to both recruitment and intervention delivery.

… in the patient who is crashing, and everything is going haywire, and they are literally about to die, again, people will say, we’ve got to do something, and REBOA is obviously an option. So, the window to actually get those patients we found where randomisation is… where patients were eligible, and REBOA is feasible is very difficult. Participant 13, trauma consultant, site 4.

### Beliefs about clinicians’ capabilities to deliver REBOA

Participants’ descriptions of past experiences of trial recruitment highlighted a few discrepancies with regards to when site staff decide to deploy the REBOA intervention. Sometimes this was linked to difficulties in judging patient eligibility (see above), which either provoked hesitancy or prompted premature decisions to randomise when the patient was subsequently perceived to no longer require REBOA. Linked to descriptions of the skill-sets required to deliver REBOA, the scarcity of eligible cases and 24/7 staff availability were comments associated with participants’ beliefs about capabilities to perform REBOA. A lack of confidence was acknowledged by clinicians who were (or would be) responsible for delivering the intervention, highlighting concerns over personal ability in a real-life setting. In addition, clinical staff who assist in the delivery of the REBOA intervention cited they had observed similar concerns in others. The concerns referenced the lack of opportunity to refine their delivery of the intervention through practice.

### Beliefs about the consequences of REBOA recruitment and intervention delivery

The majority of participants indicated that they believed the REBOA intervention could be beneficial to many patients. In addition, some participants recognised the reputational benefits associated with trial involvement, such as opportunities for emergency departments to showcase their contribution to research. Together, these beliefs motivated site staff to recruit patients to the trial.

Stuff that would encourage me is that we would be sort of upping our game in trauma by recruiting patients and by contributing to this trial. I think there’s also a bit of a reputational advantage for the department, for the Emergency Department and the trauma service to show other services that, you know, we are taking part in research even during stressful times [global pandemic] and I think that’s sort of a badge of honour. Participant 3, trauma consultant, site 6.

Many participants discussed their concerns around patient eligibility with particular reference to diagnosing exsanguinating haemorrhage. In addition, some participants indicated that people could hold different views about patient eligibility. Sometimes this could act as a barrier to trial recruitment and intervention delivery.

…I think it’s going to take quite a bit of work before we work out and we can prove how you diagnose who is genuinely exsanguinating as opposed to who is bleeding a bit… and then how you can go about predicting which patients are associated with a need for this kind of procedure and those which actually would have been alright without it. Participant 2, trauma consultant, site 4.

When considering challenges around intervention delivery, several participants acknowledged that the REBOA intervention may cause complications. Anticipation of negative side effects sometimes affected decisions to deliver the intervention. One trauma consultant suggested that the anticipatory negative consequences impact decisions to deliver the intervention as well as feelings of nervousness amongst first time operators, which is linked to beliefs about capabilities:

… so I’ve talked about worrying about side effects haven’t I and that affects your decision making to do it, I think the other thing is that I think operators would be nervous about their first time… Participant 5, trauma consultant, site 6.

### Social influences of REBOA recruitment and intervention delivery

Many participants indicated that individuals within their trauma teams often exhibited different levels of equipoise, with some members having a clear preference for either delivering REBOA (or not) to treat eligible patients. This was often linked to beliefs about patient eligibility.

…some doctors that are very on board with it and really want to try, but it’s a numbers game and I feel like if the senior doctors that have been here longer, some of them don’t like it and therefore that carries more sway than anything... Participant 8, research nurse, site 1.

Nevertheless, one participant suggested the presence of collective equipoise amongst their team, whereby a preference for REBOA delivery exhibited by some was balanced by clinicians who adopted a more cautious approach.

There was a bit of friction within the hospital in terms of whether we should be doing REBOA, who does REBOA. The trauma surgeons are quite keen that it’s not done too liberally. Many of the pre-hospital physicians are quite pro-REBOA, and I think that the discussions that happen on an institutional level bear out those differences of opinion. Participant 6, clinical research fellow, site 5.

In addition to the influences of others with regard to equipoise and its impact on recruitment, other social influences also impacted on intervention delivery. Participants cited their observations of the clinical team possessing doubts about REBOA recruitment/intervention delivery. This was attributed to their collective worry about the complications that could occur following REBOA. However, the majority of participants indicated that their trauma teams were generally enthusiastic about their participation in the REBOA trial, often expressing altruistic motivations for trial participation. Many participants also acknowledged their appreciation of the trauma-expert delivered training received as a result of their role within the trial.

It’s been a very good way for us in [hospital] to work with the trial team and access that expertise. Certainly, when they came and did the training day, it was almost less about REBOA, and more about we’ve got a couple of really top-drawer trauma experts just talking about trauma and cases for us, and the feedback for that training day was outstanding…I think there’s a number of perhaps unwitting side effects to all of this, really, in terms of generating dialogue, generating education, that is very, very important. Perhaps the trial didn’t set out to do that, but it’s achieved that. Participant 15, trauma consultant, site 8.

### Memory, attention and decision-making processes during the conduct of REBOA trial delivery

Participants’ descriptions of past experiences of trial recruitment highlighted a few discrepancies with regards to when site staff decide to deploy the REBOA intervention. Sometimes this was linked to difficulties in judging patient eligibility (see above), which either provoked hesitancy or prompted premature decisions to randomise when the patient was subsequently perceived to no longer require REBOA.

I think we got a little bit ahead of ourselves in the heat of the moment and randomised the patient. We didn’t actually, and weren’t stupid enough to put the REBOA balloon in having realised the patient probably didn’t need it. We discussed all of this at length with our [name of PI and deputy] after the event, and worked it through. Participant 7, trauma consultant, site 3.

As specified previously, REBOA is typically conducted in a fast-paced, stressful environment by clinicians who reportedly have few opportunities to master the technique outside of a simulated setting. Consequently, the actual delivery of the REBOA intervention was often reported by participants to require significant mental resources (e.g. concentration and memory).

You need the bandwidth when you’re standing at the end of the bed to get a real global appreciation of what’s going on, which is what we always encourage from trauma team leaders anyway. But if you get stuck in doing something practical or you’re helping out with the airway or a chest intervention or something, then that’s going to make life difficult for yourself. Participant 15, trauma consultant, site 8.

In addition, the dual act of considering the intricacies of randomisation, such as locating the recruitment app and the conduct of the REBOA intervention within an emergency department setting, was also considered challenging.

### Behavioural solutions: ‘treating’ the trial process problems through development and implementation of evidence-based strategies

We identified twenty-four potential BCTs that could support REBOA trial recruitment and clinical intervention delivery based on the barriers and enablers identified from the TDF diagnosis phase. Table 2 provides a detailed overview of the proposed solutions, first by behavioural solution focus (i.e. Training, Environmental Restructuring and Enablement), followed by solution content, linked BCTs, belief statements to illustrate how the interview findings informed the solution development, and the APEASE assessment. Whilst many of the identified barriers are actionable through the development of targeted solutions, it is important to recognise that some barriers (such as the need for dedicated research nurses or clinical research fellows, or a 24/7 service to deliver the REBOA intervention) were not practical within this project and talk to wider infrastructure support costs for research more generally. Therefore, these challenges were not prioritised for solution development within the UK-REBOA trial. The priority evidence-based solutions identified included a range of potential strategies. Some of these potential solutions were already active within existing trial practices (e.g. prompt sheets describing recruitment and intervention delivery targeting the memory, attention, and decision-making processes domain), but novel strategies were also identified.

**Table 2:** Proposed solutions, linked to the barriers/enablers they address and the associated APEASE criteria

Proposed solution(s)	Proposed content	Selected BCT(s) (domain-relevant/supplementary)	Belief statements (salient barriers/enablers, linked to TDF domains)	Inclusion record (including APEASE criteria)
Training	Target altruistic emotions — express satisfaction of being part of a trial which will influence clinical practice Encourage reflection of the pros/cons to recruitment in the trial generally. Including advantages of knowing which clinical method is most effective. Highlight how the research will influence clinical practice. Remind staff about the potential benefits of REBOA to patients with traumatic injury, despite the associated risks. Also benefits of not doing REBOA — standard care. The purpose of the trial is to find out which method is best. Highlight that staff are contributing to valuable research which will also benefit the reputation of each institute. Present case studies of real-life examples where patients have been treated with REBOA and standard care, and highlight the valuable contribution of the trial Link the benefit of taking part in the trial to anticipated regrets of failing to recruit eligible patients. Remind staff of the scarcity of cases. Highlight the requirement to address the trial research question	5.6. Information about emotional consequences 9.2. Pros and cons 5.1. Information about health consequences 5.3. Information about social and environmental consequences 5.2. Salience of consequences 5.5. Anticipated regret	‘Reputational benefit for the institute associated with being able to recruit patients and deploy REBOA’ (TDF Beliefs about consequences) ‘REBOA may be beneficial’ (TDF Beliefs about consequences) ‘REBOA may cause complications’ (TDF Beliefs about consequences) ‘It can be difficult to define exsanguinating haemorrhage’ (TDF Beliefs about consequences)	**Include BCTs 5.6., 9.2., 3.2., 5.1., 5.2.:** All APEASE criteria met **Exclude BCT 5.5**: May not be acceptable. Many valid reasons for not recruiting eligible patients, external, out-with control. APEASE Acceptability, Equity and Side-Effects criteria not met
Training	Include step-by-step instructions on how to recognise eligibility and perform REBOA: provide a demonstration by presenting video clips. All sites have to agree on eligibility criteria. Provide case study examples Set easy-to-achieve tasks (e.g. the areas which site staff find simple to complete, such as navigating the randomisation app) and progress to more complex steps, such as monitoring eligibility and performing REBOA	6.1. Demonstration of the behaviour 8.7. Graded tasks 8.1. Behavioural practice/rehearsal	‘Recognising an eligible patient requires expertise’ (TDF Skills) ‘Insertion of REBOA can be technical’ (TDF Skills) ‘Concerns about competency due to low throughput of cases’ (TDF Skills)	**Include** all BCTs (already delivered during on-site training): APEASE criteria met
Training	Incorporate advice on how to reduce the cognitive load of performing REBOA and randomising a patient. This can include assigning other tasks completed simultaneously to different members of the team	11.3. Conserving mental resources	‘You need to remember technical aspects of REBOA’ (TDF Memory Attention and Decision Processes) ‘Our team is inclined to wait to see if our patient requires REBOA’ (TDF Memory Attention and Decision Processes)	
Environmental restructuring	Social prompt: Assign an individual to prompt REBOA randomisation/delivery when a code red is flagged. This could include prompting eligibility assessment or technical aspects of REBOA. Remind healthcare professionals of the protocol. Encourage the use of memory aid sheets to facilitate memory of REBOA recruitment and the procedure. Can include provision of cue cards to be slotted into staff lanyards Sites could purchase a mannequin/or recycle use of existing mannequin to practice REBOA on a weekly basis. Arrange for colleagues to provide practical help to recruitment and delivery of REBOA in each shift. This may include providing contact details of those who can help during out-of-hours Assign REBOA champion roles at each site and highlight support available during team meetings Ensure staff have a device with the app readily accessible for randomisation and gather essential equipment or prepare a REBOA trolley to assist in the delivery of the intervention This could also include a diagram of the ideal positioning of staff during a code red call	7.1. Prompts/cues 12.5. Adding objects to the environment 3.2. Social support (practical) 12.2. Restructuring the social environment 12.5. Adding objects to the environment 12.6. Body changes 12.1. Restructuring the physical environment	The clinical context for REBOA is inherently stressful and fast-paced (TDF Environmental Context and Resources) ‘There are so few patients who require REBOA’ (TDF Environmental Context and Resources) ‘The ability to recruit depends on staff availability’ (TDF Environmental Context and Resources)	**Include** all BCTs: APEASE criteria met. Whilst some BCTs were already incorporated in trial practices, it was recommended that delivery of all BCTs should be monitored to ensure continuous implementation
Enablement	Encourage staff to praise local efforts of recruitment and REBOA delivery when applicable. Praise can also be communicated via Email, as well as during local PI meetings Encourage sites to provide monthly updates on the progress of REBOA trial recruitment and intervention delivery during trial meetings. Facilitate detailed discussion about recruitment procedures: ask staff to provide a description of the latest recruitment cases including ‘near misses’ (when applicable). CIs to provide information about whether they approve of the procedures/decisions adopted Prompt discussion of what went well and what might have been done differently. Include action plans to tackle similar situations in the future Maintain the enthusiasm of REBOA by advising staff to encourage others to recruit and randomise eligible participants See examples listed above 5.3.: designed to target mixed levels of team equipoise (beliefs about the consequences of REBOA intervention delivery). Delivered as bespoke infographic to be distributed to all site staff. Provide contact details of Clinical CI and Clinical Lead: highlight support available	10.4. Social reward 6.3. Information about others’ approval 3.2. Social support (practical) 6.2. Social comparison 1.2. Problem solving 1.4. Action planning 3.1. Social support (unspecified, practical) 5.3. Information about social and environmental consequences 3.2. Social support (practical)	‘Our team is enthusiastic about the REBOA trial’ (TDF Social influences) ‘People can hold different views about patient eligibility’ (TDF Social influences) ‘Our team has mixed levels of individual equipoise’ (TDF Social influences, TDF Beliefs about Consequences)	**Include**: all APEASE criteria met Whilst some BCTs were already incorporated in trial practices, it was recommended that delivery of all BCTs should be monitored to ensure continuous implementation
Persuasion Enablement	Remind staff that they have successfully performed REBOA and recruited participants in simulation and/or in real life Enabled by PIs Local principal investigators (PIs) can actively persuade relevant staff members that they are capable of performing the REBOA intervention during conversations/meetings. Highlight transferable skills of trial recruitment — include the successful past experience of trial involvement Encourage staff to practice positive self-talk as a team: this could include discussing one’s own achievements/successes in a group setting. PIs to deliver	15.3. Focus on past success *can also be incorporated into training 15.1. Verbal persuasion about capability 15.4. Self-talk	‘Clinicians have to be confident to deliver REBOA; this can influence recruitment’ (TDF Beliefs about capabilities) ‘There is lots of nervousness around delivering REBOA related to personal abilities’ (TDF Beliefs about capabilities)	**Exclude**: Difficult to implement. Depends on factors less amenable to change – e.g. PI personality and workplace culture BCTs 15.3. and 15.1. can instead be incorporated via trial Training practices APEASE Effectiveness criteria not met APEASE Practicability criteria not met for BCT 15.4. Difficult to implement in a trauma care setting

One of the potential solutions to address several barriers was adaptations or updates to *trial training* packages. The behavioural investigation identified the need to target aspects already covered by the site training. For example, step-by-step instructions on how to randomise patients and perform REBOA (BCT Demonstration of the behaviour). Novel areas to target included the impact of altruistic emotions by highlighting staff contributions to valuable research that could change clinical practice (BCT Information about emotional consequences), and reminding staff they have successfully recruited participants and/or performed REBOA in simulation or real-life (BCT Focus on past success). Both BCTs were incorporated within a verbal discussion during simulation-based training.

**Fig. 2:**
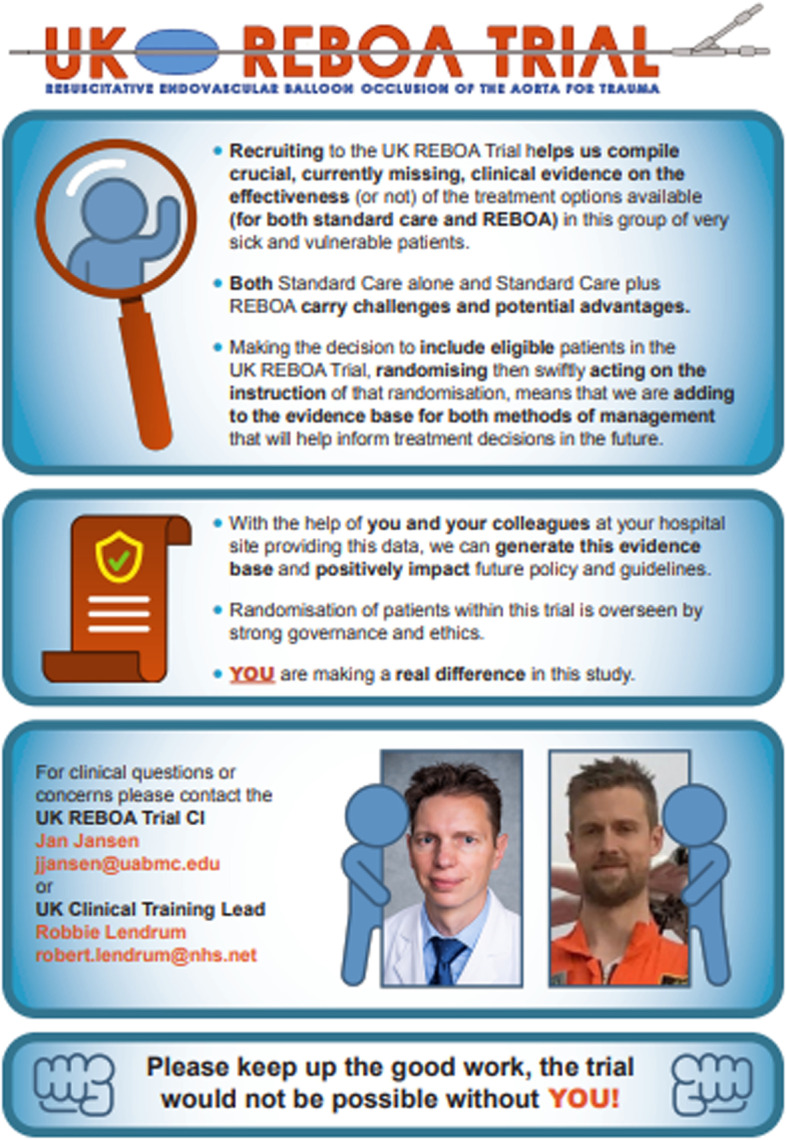
Bespoke infographic containing BCTs

We also identified the requirement for solutions that could be applied to *restructure the physical and social environment* and enact other processes and procedures to *enable* recruitment and intervention delivery. One of the solutions developed to potentially address the relevant barriers was a bespoke infographic (see Fig. 2) designed to target variations in individual equipoise amongst trauma teams (TDF Social Influences, Beliefs about consequences). The infographic contained BCTs that reinforced the purpose of the trial with information about the social and environmental consequences of recruitment/REBOA intervention delivery (BCT Information about social and environmental consequences), as well as contact details of the clinical co-CI and clinical training lead to indicate the support available (BCT Social support, practical). The infographic was distributed by the trial office to all site staff involved with recruitment (provided by email) and was requested to be shared amongst other site staff involved in the trial (paper copies for sharing).

Other potential solutions proposed, based on interview findings, included upscaling the use of training mannequins to facilitate skills acquisition/maintenance via simulating trial recruitment/intervention delivery (BCT Adding objects to the environment). Staff were encouraged to share mannequins across sites to facilitate rehearsal of the REBOA procedure and recruitment processes. Also, the development of a single-page de-brief proforma used as a learning tool for staff following a randomisation (or sometimes missed randomisation) to share experiences of trial processes enacted (or not) including anonymised case details about patient eligibility and procedural descriptions of recruitment/intervention delivery (BCTs Social support (practical) and Social comparison). The proformas could prompt staff to proactively plan for any events that may occur unexpectedly on the basis of their past experiences, as well as consider solutions to overcome challenges that may arise in the future (BCTs Action planning and Problem solving). This information would be collected by the trial team (likely the trial manager or clinical co-CI) during communication (ideally a call with all involved in the case at site) to encourage discussion and reflection. See Table 3 for a sample list of questions including BCTs. Findings also supported the ongoing praising of staff for their efforts in recruitment and intervention delivery when applicable (BCT Social reward). Praise was often communicated to sites via Email or Twitter following a randomisation. See Table 2 for a list of proposed solutions, linked to the barriers and enablers they address.

**Table 3:** A sample list of questions including BCTs

•How did you identify patients eligible for REBOA?
•Can you provide step-by-step information regarding the procedures you followed before/after randomisation?
•What were the challenges you faced during this case?
•Which aspects of recruitment/intervention delivery went well? Why?
•Is there anything you would do differently if a similar case arose in the future? (can you think of any solutions?)

## Discussion

Our study aimed to apply an innovative methodological approach to identify behavioural trial process problems relevant for recruitment and clinical intervention delivery and propose targeted, theory-based, evidence-informed solutions to potentially improve trial processes. We used the UK-REBOA trial as a case study in this proof-of-concept approach to explore the feasibility of diagnosing and treating specific challenges that affected key trial behaviours (recruitment and REBOA intervention delivery).

To the best of our knowledge, this is the first study that has adopted a behavioural approach to examine the factors, and develop potential solutions, that influence recruitment and intervention delivery in a trauma trial setting. A recent qualitative study explored clinician-reported challenges to recruitment in trials set within emergency care and identified a range of influential factors, including supporting the patients to engage with the research, issues around equipoise, surgeon preferences, interpretation of eligibility criteria, and balance of clinical vs. research roles [8]. In addition, other recent studies have incorporated behavioural frameworks to identify challenges for trial recruitment and intervention delivery [3, 4, 10, 11]. However, the majority of these existing studies do not go as far as developing potential solutions and implementing them within the trial to proactively address the challenges identified. One study has emphasised the utility of a behavioural approach to develop solutions targeting oncologists designed to combat recruitment issues of rural patients in urological cancer trials [23]. Systematic incorporation of evidence-based theory-informed techniques designed to mitigate identified challenges to effective trial practice may be more likely to achieve desired change compared to atheoretical approaches to solution development, as has been demonstrated in clinical practice [24].

The findings presented in this paper suggest that some domains within the TDF did not appear to be as relevant with regard to behaviours important for REBOA recruitment and intervention delivery — such as Social/Professional Role and Identity and Behaviour Regulation. These domains have been shown to affect recruitment within trials in other clinical settings [4]. For example, TDF studies that examined barriers and enablers to recruitment within trials set in elective care have suggested that reminders are helpful to enable discussions around recruitment to patients (Behaviour Regulation) [4]. In addition, the impact of the Social/Professional Role and Identity domain has been illustrated in other recruiters’ accounts of how recruitment in clinical trials feels like an integral part of their professional identity [4]. These results highlight that context likely plays a key role in influencing the range of challenges experienced by trial teams in successful trial delivery. As more studies use behavioural approaches to understand trial recruitment and retention, the potential for aggregative assessments of findings from studies using similar data tools will also be realised. The synthesis of findings across clinical contexts and clinical intervention types (and many other variables) could contribute significantly to the generation of transferable strategies targeting notable process problems within clinical trials.

Our study also highlights the value of this behavioural approach to adapting (or in future informing initial development of) trial training to optimise and incorporate theoretically informed solutions to address ‘live’ recruitment and intervention delivery challenges identified during the conduct of a trial. This behavioural approach, using the TDF, has demonstrated its utility to develop a behaviour-based implementation strategy for trauma team training in clinical care rather than a trial context [7]. Our study suggests that trial training also provides an ideal opportunity to incorporate theoretically informed strategies to alleviate multiple challenges that may affect recruitment/intervention delivery.

The next steps would be to determine whether this approach translates to demonstrable improvements in recruitment and/or intervention delivery. This will likely need a trial somewhat larger than the UK-REBOA trial, given its low throughput of eligible patients. However, other markers of ‘success’ can be measured which could include perceptions of the relevance of the training and site staff confidence in delivering the trial.

### Strengths and limitations

Our study demonstrates that the incorporation of a behavioural approach to understanding trial processes provided practical advantages: understanding the underlying determinants that affect behaviour, attitudes and beliefs in a clinical trial provided an avenue to implement theoretically informed evidence-based solutions to potentially enhance trial practices. Although the effectiveness of the solutions we propose remains to be tested, we have demonstrated the proof of concept to this approach which can be used to inform the conduct of process evaluations within other clinical trials.

A potential limitation could be our use of two separate interview topic guides. However, during the analysis, common TDF-based themes were identified throughout all of the interviews. This demonstrates the flexibility and relevance of applying the TDF within the analysis process when the interview questions may/may not be guided by the theoretical domains. Furthermore, our study also raises an interesting methodological question about whether TDF-based topic guides actually facilitate more in-depth responses compared to interview questions that are not designed to cover the theoretical domains [25]. Future studies could address this issue.

## Conclusion

This study demonstrates how a behavioural approach can be applied to address trial conduct challenges, namely, difficulties for recruitment and the delivery of complex interventions within a trial. Through using this approach, the influences on behaviours, attitudes and beliefs were diagnosed and treatments to optimise these were developed. These treatments now require formal evaluation to determine the effectiveness of this approach.  

## Supplementary Information

**Additional file 1:** Depiction of the REBOA procedure

**Additional file 2:** Phase1 Interview Topic Guide

**Additional file 3: **Phase 2 Interview Topic Guide 

**Additional file 4: **Table of TDF domains and belief statements. Provides a summary of the content within all TDF domains, with example quotes andassociated frequency data 

## Data Availability

No data is available for sharing beyond those published.

## Abbreviations

REBOA: Resuscitative endovascular balloon occlusion of the aorta
TDF: Theoretical Domains Framework
TTL: Trauma team leader
BCT: Behaviour Change Technique
BCW: Behaviour Change Wheel
APEASE: Acceptability, Practicability, Effectiveness, Affordability, Side effects, and Equity
