# Supplementary material for: Behavioural optimisation to address trial conduct challenges: case study in the UK-REBOA trial
Source: Trials. 2022 May 12;23:398. doi: 10.1186/s13063-022-06341-6 (PMC9097042; doi:10.1186/s13063-022-06341-6)
Supplement: Supplementary file 1 — Additional file 1: Depiction of the REBOA procedure [file 13063_2022_6341_MOESM1_ESM.docx]

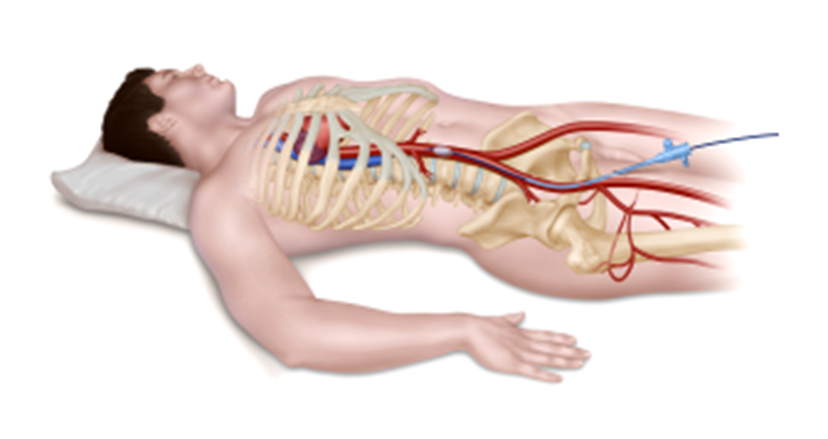


Depiction of the REBOA procedure.

The balloon can be deployed in the descending thoracic aorta (referred to as “zone I”) or the distal


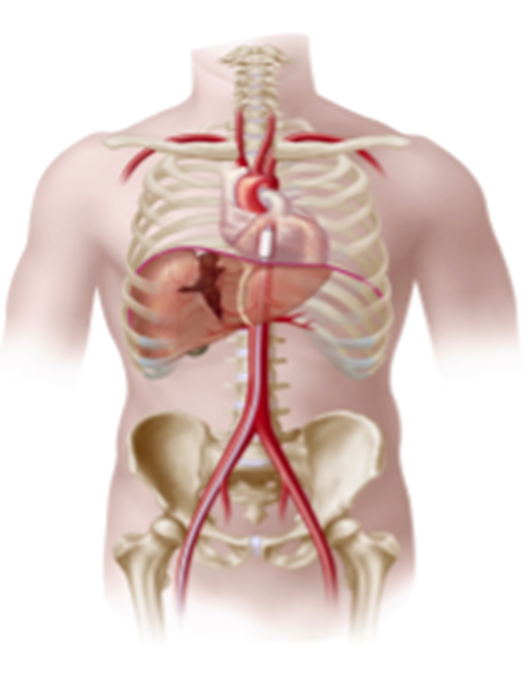

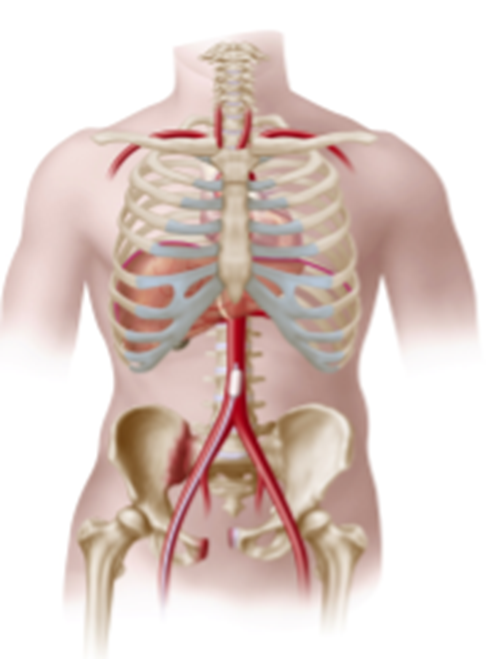


The REBOA balloon inserted in the distal abdominal aorta (referred to as “zone III”).

The REBOA balloon inserted in the descending thoracic aorta (referred to as “zone I”).
