# Supplementary material for: Behavioural optimisation to address trial conduct challenges: case study in the UK-REBOA trial
Source: Trials. 2022 May 12;23:398. doi: 10.1186/s13063-022-06341-6 (PMC9097042; doi:10.1186/s13063-022-06341-6)
Supplement: Supplementary file 2 — Additional file 2: Phase1 Interview Topic Guide [file 13063_2022_6341_MOESM2_ESM.docx]

**Phase 1 Interview Topic Guide**

**Discussion and Signing of the Consent Form:**

- Thanks for returning the consent form.
- So, the purpose of the interview today is really for me to explore your experiences and views about the UK REBOA trial – we’re interested here in local clinical team member’s experiences/ views particularly around the recruitment and consent processes– so please do feel free to express what you think.
- Ask if there are any questions or concerns.

| Topic | Questions and prompts |
| --- | --- |
| Explore role in general and specifically in relation to the UK REBOA trial. | How long have you been involved with REBOA/what is your role within the trial? |
| Explore decision to become involved in the trial. | Where did you learn about the trial?  Did you have a particular interest in the topic of the research?  Why did you decide to participate? |
| Explore views about the rationale of the UK REBOA trial. | How would you describe the purpose of the trial?  Can you tell me what you understand about the evidence relating to REBOA?  What do you personally think about the REBOA procedure?  Which patients are eligible? Do you think that’s correct/sufficiently helpful (incl criteria)? Are there patients who you think are perhaps missed (why)? How do you define/operationalise exsanguinating haemorrhage?’  Are there any patients who are not included? |
| Explore views about the planned recruitment process at their hospital. | Can you tell me who is involved?  What are their roles specifically relating to recruitment?  Can you talk me through the patients’ recruitment journey at your hospital?  Are there any barriers which impact on recruitment (organisational/personal)?  If barriers identified, explore what they think could help overcome these barriers? (i.e. can you suggest any potential solutions)  Are there any aspects which facilitate recruitment? (organisational/personal – also probe for views about the dedicated and secure website for randomisation accessed via tablets/smartphones) |
| Explore views about the consent process. | Views about the initial recruitment process – randomisation needs to take place without consent – how do you (your colleagues) feel about that/how does that work at your hospital? What if family members are there at the time?  So, REBOA involves research without prior consent - Views about the consent process – how does that work at your hospital (explore for both patients who have capacity, as well as those patients who lack capacity)  Views about patients who die before subsequent consent can be obtained (NB: this is consent to use data as intervention will have already happened) REBOA supports the use of ‘passive’ methods to inform relatives of the patient’s involvement – what do you understand/think about this? |
| Broaden discussion out for last part of interview, exploring views on REBOA more generally (i.e., identifying any perceived barriers/facilitators, both locally and nationally, to the introduction of REBOA). | What have you learnt since you started the trial?  What advice would you give to new sites about to start? |

**Explore any other issues of relevance to participant not covered by the above before wrapping up discussion.**

**Thank for participation in the study.**
