# Supplementary material for: Behavioural optimisation to address trial conduct challenges: case study in the UK-REBOA trial
Source: Trials. 2022 May 12;23:398. doi: 10.1186/s13063-022-06341-6 (PMC9097042; doi:10.1186/s13063-022-06341-6)
Supplement: Supplementary file 3 — Additional file 3: Phase 2 Interview Topic Guide [file 13063_2022_6341_MOESM3_ESM.docx]

**Phase 2 Interview Topic Guide**

*This topic guide will be used in a flexible manner to generate discussion between trial staff interviewees and the researcher. This topic guide may be subject to refinement.*

**Inform participants that:**

My name is X, and I am a researcher at the University of Aberdeen. Thank you for agreeing to participate in this interview. The general aim of the study is to help us understand more about the experience of recruiting participants into trials. **We want to talk to you about the barriers and facilitators to recruiting participants - with a specific focus on the UK-REBOA trial.** Just to refresh your memory – the UK-REBOA trial is a multi-centre patient randomized trial. It is currently on-going - and aims to test if REBOA in addition to standard major trauma centre treatment is more effective than standard care alone.

I have a number of questions I’m going to ask you - and I’d like you to give them some thought and answer frankly. **We are interested in your experiences and views so there are no right or wrong answers.** **All information collected will be strictly confidential.** **Our chat today will be recorded. The audio tapes will only be used for transcribing and analysing data.**

**How does that sound?**

**Do you have any questions for me before we start?**

| 1. Do you identify as male or female? |
| --- |
| 1. What trial site do you work from? |
| 1. What is your job title? |
| 1. How long have you been a (job title)? |

**Do I have your consent to get started?**

| Question | Prompt |
| --- | --- |
| 1. Can you tell me about your role in recruitment of participants - pertaining to UK-REBOA trial? |  |
| 1. Can you describe the steps involved in recruiting participants for UK-REBOA trial? |  |
| 1. Do you use any protocols, policies or guidelines to guide your discussions for trial recruitment? | If so, which ones? and what about for the delivery of the intervention? |
| 1. Do you think (the protocols, policies or guidelines) are useful? | For recruitment and intervention delivery (if used) |
| 1. (If not) Is there any reason that you do not use guidelines? | Do you think guidelines are a waste of time? |
| 1. How easy/difficult is it to successfully recruit participants for UK-REBOA trial? |  |
| 1. What skills are required to recruit participants for UK-REBOA trial? and what about for the delivery of the intervention? | Knowledge of evidence and discussion about potential harms/benefits |
| 1. Have you ever had any formal training in recruiting participants into UK-REBOA trial? |  |
| 1. To what extent do you see recruitment for trials as part of your current professional role? | What are those roles |
| 1. How confident are you in recruiting participants for UK-REBOA trial? and what about for the delivery of the intervention? |  |
| 1. What problems/difficulties do you encounter when recruiting participants for UK-REBOA trial? And what about for the delivery of the intervention? |  |
| 1. What would help you overcome these problems/difficulties? |  |
| 1. Overall, do you expect eligible participants to be recruited into the UK-REBOA trial? | Why are you optimistic/not optimistic?  Have your expectations changed over the course of the trial? |
| 1. In your opinion, what are the benefits and downsides of recruiting participants to UK-REBOA trial? | to yourself, to the patients i.e. what harms might be avoided? colleagues, healthcare Organization NHS – positive & negative, long/short-term and financial |
| 1. What would happen if you were unable to successfully recruit participants to UK-REBOA trial? |  |
| 1. Have past experiences influenced how you discuss UK-REBOA trial recruitment with potential participants? |  |
| 1. Is there anything that encourages or discourages you to consider potential participants for the UK-REBOA trial participants? | any rewarding and unrewarding incentives, the team gets recognized/gold star |
| 1. In what situations do you find yourself more or less motivated to recruit potential participants for your UK-REBOA trial site? | Why? |
| 1. How important or unimportant you feel it is to recruit potential participants for UK-REBOA trial? | how high is the priority in relation to other aspects of your role? |
| 1. Are there specific targets or quotas you need to meet? |  |
| 1. How do you decide which patients to recruit to the UK-REBOA trial? | Does memory play a role? Is it possible to forget to talk to participants about UK-REBOA trial?) |
| 1. Can you give me an idea of the kinds of situations that arise where it becomes difficult to consider potential participants for the UK REBOA trial? | uncertainty about if the participants are right for the trial, or how ill the patient looks |
| 1. Are there specific aspects of the work environment that make it easier or more difficult for you to recruit patients for UK-REBOA trial? and what about for the delivery of the intervention? | patient factors, colleagues, recourse issues, equipment, less competing tasks or time constraints? |
| 1. To what extent are resources for UK-REBOA trial recruitment available at your trial site? |  |
| 1. How does the views of other colleagues affect your approach in considering patient participation in UK-REBOA trial? | what about other clinicians; medical staff including nurses and residents/fellows; relatives of the participants etc. |
| 1. Do others’ emotions affect discussions or decisions about recruitment to the UK-REBOA trial? |  |
| 1. Can you describe how you feel when you recruit patents into the UK-REBOA trial? (E.g. emotions such as guilt, worry, concern or satisfaction) | How do these emotions influence the discussion or the decision? |
| 1. How do you think emotions, or tense situations at your site influence how you recruit UK-REBOA trial patients? |  |
| 1. Are there strategies you currently use to assist UK-REBOA trial recruitment process? Is there anything you would alter – concerning how you recruit potential participants? and what about for the delivery of the intervention? | Formal or informal strategies, or personal methods? Can you think of any recent examples?  What would you do differently then? Are there any other strategies or ways of working you might try? |
| 1. Do you receive any feedback about your recruitment? What form does that take? What about for the delivery of the intervention? | In what form? Does that change your behaviour (or does this make a difference?) |
| 1. Last question! How do you think current NHS circumstances, in terms of set-up and response to COVID-19, influences recruitment to the trial? What about the delivery of the REBOA intervention? |  |
| 1. That’s all the questions I have for you, is there something else you’d like to say or expand on? |  |

**Explore any other issues of relevance to participant not covered by the above before wrapping up discussion.**

Thank you very much for your time
