# Supplementary material for: Behavioural optimisation to address trial conduct challenges: case study in the UK-REBOA trial
Source: Trials. 2022 May 12;23:398. doi: 10.1186/s13063-022-06341-6 (PMC9097042; doi:10.1186/s13063-022-06341-6)
Supplement: Supplementary file 4 — Additional file 4: Table of TDF domains and belief statements. Provides a summary of the content within all TDF domains, with example quotes andassociated frequency data [file 13063_2022_6341_MOESM4_ESM.docx]

| Domain (frequency out of 18) | Belief Statements (frequency out of 18) | Example Quotes |
| --- | --- | --- |
| Knowledge (17) | I am aware of the recruitment processes in REBOA^[[1]](#footnote-1)^ (16) | *Yeah. It works okay because obviously we can’t get the consent of the patients whilst they’re in critical care which I think is fair and then we tend to get professional consent as well from one of our representatives which is one of our senior nurses who’s not involved in the study and then we’ll try and get relative or patient consent if possible. But yeah, it works fine. Participant 1, Research Nurse, Site 6* |
|  | I am aware of the patient eligibility criteria for REBOA^1 2^(8) | *Yeah. I think the criteria are correct, actually. I think the key word is exsanguination. The indication, if you read them verbatim, talk about exsanguination, the definition of exsanguination is bleeding to death. Participant 2, Trauma Consultant, Site 4*  *I would define it as a haemodynamic instability which is usually by blood pressure although there might be other clinical signs after at least pack one of… described as pack one of the massive haemorrhage protocol which I think is similar in most other hospitals which should be four units of blood, four units of FFP. I think that’s a sufficient enough blood volume resuscitation to regain your haemodynamic stability. Participant 3, Trauma Consultant, Site 6* |
|  | I am aware of the existing evidence on the use of REBOA (10)^2^ | *As far as I’m aware mostly the evidence for REBOA comes from animal studies and then retrospective observational studies. And, so I think there might also be one at this stage kicking off in terms of RCT, but as far as I know this is the only randomised controlled trial of REBOA. And, so yeah, the evidence is limited by all those transitional and retrospective…things that you have with this kind of study. So yeah, I think that’s kind of the main research. Participant 4, Clinical Research Fellow, Site 4*  *So specifically for the patients, but there isn’t enough evidence otherwise we wouldn’t be running the trial, so there is that point around emergency department REBOA or sort of frontline REBOA. Participant 5, Trauma Consultant, Site 6* |
|  | I know the steps involved in enacting the REBOA intervention (8)^2^ | *My understanding of a few patients that are randomised, and certainly we’ve done it here, is that you assess the patient, it’s clearly a dynamic process, but in the time between assessing them, then randomising, and then inflating the balloon, it might take you ten minutes to actually deploy the intervention. Participant 6, Clinical Research Fellow, Site 5* |
| Skills (17) | Insertion of REBOA can be technical (9)^2^ | *Not in the trial, but we have had problems with balloons going up into the vena system or just bad things that you wouldn’t want to happen, probably because people have been trained a bit, but not to a level that they’re comfortable with it. Participant 6, Clinical Research Fellow, Site 5*  *People like me do a lot of central lines, so actually, the methodology per se isn’t hugely different, and is a transferrable skill. Participant 7, Trauma Consultant, Site 3* |
|  | Recognising an eligible patient requires expertise (5)^1 2^ | *P -* *I think it takes a lot of skill to know if they’re amenable to REBOA, so to confirm eligibility, I think that takes a lot of skill, and I think the insertion itself, yeah. Participant 8, Research Nurse, Site 1*    *I think you need the generic professional skill of recognising what a critically sick bleeding patient looks like, but that skill I would say is common, by then it could always be refreshable and refresh, but it’s common to the skill set of people working on the front line in modern trauma care, so ED positions and trauma anaesthetist. Participant 9, Anaesthetist, Site 8* |
|  | Concerns about competency due to low throughput of cases (9)^2^ | *It’s hard, it’s hard to train enough people to do the procedure and because the numbers are low it’s hard to maintain the skills. Participant 2, Trauma Consultant, Site 4*  *No, I do think that that’s something down the line, but I think ultimately the issue is going to be numbers and maintaining training competencies in a system that less than a third inclusion criteria come much reduced. You know maintaining competence. Participant 10, Trauma Consultant, Site 3* |
| Social Professional Role & Identity (17) | My team role influences my decisions to recruit participants to REBOA (13)^1^ | *Absolutely and again I think that’s not the sort of thing even as team lead that’s not the sort of thing that I’m going to making in isolation, I will be talking to the critical care doctors who are there, the surgical team that are there, and that will be a joint decision. But I mean albeit that will be a decision taken in a fairly short period of time. Participant 12, Trauma Surgeon, Site 4*  *I’m part of the trauma team, a trauma surgeon. So, when the trauma call came in, we attended the trauma call, I provided the initial surgical opinion. Participant 11, Trauma Surgeon, Site 7* |
|  | My research interests encourage me to take part in REBOA (6)^1 2^ | *Yes. My main interest is major trauma, particularly major haemorrhaging, and having an interest in that area and I hadn’t done any research myself in terms of leading on it that the PI for another trial prior to that, and I’d been involved (inaudible) prior to that, so I’ve got an interest in that area, so leading on this was just a natural progression really. Participant 10, Trauma Consultant, Site 3*  *Yes, I was motivated because I’m a [role], and [institution] has got a fairly strong history of trying to develop REBOA, and I was given the opportunity to lead on the trial from a research side of things, and so I took it in order to learn a bit more about it and understand its strengths and weaknesses. Participant 6, Clinical Research Fellow, Site 5* |
|  | My individual role influences decisions about recruitment and delivery of REBOA (4)^1 2^ | *I think as a PI and for somebody who’s motivated to get people recruited into the trial, I’ve probably got to be aware of my own bias, but actually my keenness and my motivation isn’t to do with REBOA, it’s to get someone into the trial, so you’ve got to kind of keep a bit of a running check on yourself about that. Participant 9, Anaesthetist, Site 8*  *To what extent do you see recruitment for trials as part of your professional role?*  *P - A lot of it. Yeah, it’s probably my main job is recruit and then along with it is the consent et cetera, but yeah, I would say it’s my primary job. Participant 8, Research Nurse, Site 1* |
| Memory, Attention and Decision Processes (17) | Our team is inclined to wait to see if our patient requires REBOA (14)^1 2^ | *What we did was, we inserted the arterial, the short arterial line that you would then put a wire back down (few inaudible words), but we didn’t put the big sheath in, we held back. So, all we actually did was what we said we would do for all of the code red patients, we would put in a femoral arterial line, regardless, and if they needed randomising, then randomising, and then if they needed REBOA then dilating up from that. I think we got a little bit ahead of ourselves in the heat of the moment and randomised the patient. We didn’t actually, and weren’t stupid enough to put the REBOA balloon in having realised the patient probably didn’t need it. We discussed all of this at length with our [name of PI and deputy] after the event, and worked it through. Participant 7, Trauma Consultant, Site 3*  *So for me when I’m choosing for these interventions, I’ll be honest and say I probably hesitate a little bit because I want to make sure that they don’t fall into the (inaudible) group that (inaudible) want them to be patients that stabilise out with a bit of a blood or that we can avoid using REBOA on. Participant 5, Trauma Consultant, Site 6* |
|  | You need to remember technical aspects of REBOA (8)^2^ | *I was fortunate enough to have been trained elsewhere in how to do it and I think that may have been part of a very standardised training package with lots of supporting literature around in terms of study and stuff that you had to learn and memorise in order to have you successfully signed off as being able to deliver the intervention and I think having come with that it took away some of the barriers to having never done something before. Participant 9, Anaesthetist, Site 8*  *So my colleague, [Name], he’s been… worked in A&E for a couple of years, but he’s like, “I forget things really easily, I learn by doing and if I’ve done it it’s really hard for me to remember it”, and I’m like, “Okay, that’s fine”. So I try and go through like practically even just showing the thing, the balloon. But my approach, I would much rather REBOA, potential REBOA come in when I am here, but that goes without saying. Participant 8, Research Nurse, Site 1* |
| Environmental Context and Resources (17) | The ability to recruit depends on staff availability (15)^1^ | *I think having a research nurses there most of the time would be a great benefit to screening these patients. Participant 3, Trauma Consultant, Site 6*  *So we… the [site] took the randomisation away from the trauma team leaders and gave it to us as clinical research fellows because we have a 24/7 staffing rota so we’re always there, and we’re recruiting to other trials. Participant 4, Clinical Research Fellow, Site 4* |
|  | There are so few patients who require REBOA (14)^1 2^ | *“I think another difficulty with this group of patients, is we’re looking at the absolute tip of the iceberg, in terms of the severity of trauma patients, so it’s relatively rare that patients are that sick. It might be 5% of all of them – the code red patients. The code red patients at [hospital], which I think is pretty busy, we’ve got maybe four or five a week. You’re talking about an event that happens maybe once a month, maybe less.” Participant 6, Clinical Research Fellow, Site 5*  *I think the [site], we do see a slightly different demographic of patients, we see a lot of the very sick patients, and I think the tolerance and the threshold is just that bit higher. Participant 4, Clinical Research Fellow, Site 4* |
|  | Many patients receive REBOA in a prehospital environment (6)^2^ | *… we’re more likely to see zone 1 pre-hospital REBOAs, and I think we’ve had a few pre-hospital. Participant 8, Research Nurse, Site 1*  *I think the sort of patients we are looking at, usually would have been dealt with pre-hospital by a (inaudible) type team, who they themselves are putting in REOBA at the roadside. We would expect to get more patients with REOBA already in them on arrival, as well. At the moment that is being done ad hoc, by people at London, helicopter ambulance, and a few others. Participant 7, Trauma Consultant, Site 3* |
|  | The clinical context for REBOA is inherently stressful and fast-paced (6)^1 2^ | *You’ve just got to say, looks like a REBOA, potential REBOA patient, let’s randomise them and then you randomise them to whatever you randomise them to, so I guess tempo influences the rapidity with which you need to make those decisions is an emotional challenge, but it’s not emotional in the true sense of the word emotional, it’s more one of those factors that conspires with you or against you to make the situation slightly more difficult than it would be if the trial wasn’t there or the option of REBOA, the potential option of REBOA wasn’t there. Participant 9, Anaesthetist, Site 8*  *On the flip side, in the patient who is crashing, and everything is going haywire, and they are literally about to die, again, people will say, we’ve got to do something, and REBOA is obviously an option. So, the window to actually get those patients we found where randomisation is… where patients were eligible, and REBOA is feasible is very difficult. Participant 13, Trauma Surgeon, Site 4* |
|  | The epidemiology of patients is complex (1)^1 2^ | *It’s just that it’s such a complex intervention, such a complex … well, it’s such a complex patient population and it’s a relatively complex intervention. I think that’s why it proves challenging. Participant 13, Trauma Surgeon, Site 4* |
| Social Influences (17) | Our team has mixed levels of individual equipoise (14)^1 2^ | *There was a bit of friction within the hospital in terms of whether we should be doing REBOA, who does REBOA. The trauma surgeons are quite keen that it’s not done too liberally. Many of the pre-hospital physicians are quite pro-REBOA, and I think that the discussions that happen on an institutional level bear out those differences of opinion. Participant 6, Clinical Research Fellow, Site 5*  *And [name] really has said to us, “If the clinicians are thinking REBOA, if they’re thinking REBOA then randomise. But if they’re not thinking REBOA then we’re not going to randomise at all”. So yeah, I think that’s what it’s mainly makes it difficult to get them into the trial. And a little bit frustrating as a purely research side is when we go down there and say, “What do you think about this patient? Can we put them into REBOA and the trial?” which happens probably once every three weeks I would say, probably a little more often than once a month, and the usual answer is, “No, they’re not sick enough and, so I don’t want to get the randomisation side that says REBOA and therefore I’m not going to do it”. “ Participant 4, Clinical Research Fellow, Site 4* |
|  | Our team is enthusiastic about the REBOA trial (12)^1 2^ | *So individuals, so when the UK REBOA trial started at our centre there was quite a lot of excitement about it because it is a new intervention, because it is very, what’s the word, fashionable I guess, so in the, in the more formal(?) literature a lot, so (inaudible) and it’s kind of in social media a lot. So I think there was a level of excitement when they opened the trial, which mean people were like, “Come on, we’re going to recruit to REBOA, it’s really cool”. So if you done a trial about old people and falls prevention there might not be quite as strong an uptake as there can be a procedure that’s about saving lives and mainly affects younger people, again, there’s sort of an element of excitement around it I think. Participant 5, Trauma Consultant, Site 6*  *Yes, the REBOA team came to [hospital] and introduced it and recruited a trauma team to take part in the trial. We were quite positive about it so that’s why we were involved. There was discussion on the merit of REBOA at the introduction, but I think everybody, at the end, thought it is an adjunct to our treatment plan and it would be something that we would like to pick up. Participant 11, Trauma Surgeon, Site 7* |
|  | People can hold different views about patient eligibility (7)^1 2^ | *Clinicians, really experienced clinicians are having a conversation with one of the [location] docs about REBOA basically the other day, and they just said, “No, they’re just not at that point where I’m thinking about REBOA yet”. So I don’t know if were missing them… as they’re all getting screened what is happening is, is that threshold is just… their tolerance is very high. Participant 4, Clinical Research Fellow, Site 4*  *I was the first one to arrive next to the patient and after that we got two trauma consultants coming in, two A&E consultants coming in, and they are both REBOA operators, and then between the three of us, we discussed -- we ticked all the boxes, and said yes, it is a REBOA patient, let us randomise. Participant 14, Research Nurse, Site 3* |
|  | Clinicians worry about the complications of REBOA (6)^2^ | *And I think that is the issue with this thing of REBOA is, so in that scenario… that patient was kind of maintaining his blood pressure and so on, so people are very reluctant, particularly given we have had significant concerns about complications from REBOA to put in a balloon, but put that balloon up at that stage, people are reluctant to do that. Participant 13, Trauma Surgeon, Site 4*  *So it’s trying to change attitudes, and I think you know, the clinicians believe these with obviously good reason, but I think it is very difficult, yeah, mostly because it is still… the complications of inserting a REBOA have… can have such dramatic effect, but I think it does, I don’t want to use the word scared, but yeah, people aren’t inclined to use it. Participant 8, Research Nurse, Site 1* |
| Beliefs about Consequences (16) | REBOA may be beneficial (12)^2^ | *I think the REBOA procedure itself would save a few lives. I’m not sure whether you would ever find a statistical difference between because we are fighting for very few numbers. I suspect that the main benefit would be in using partial REBOA because it gives you a bit more time at least from what I’ve read from the very basic evidence there is. Participant 3, Trauma Consultant, Site 6*  *So now we’re looking at other techniques that may help improve survival in these similar groups of patients, so REBOA is the most promising of those techniques where, well we maybe still have uncertainty in whether or not it will result in beneficial outcomes for those patients, so but it seems like a sensible approach. Participant 12, Trauma Surgeon, Site 4* |
|  | REBOA may cause complications (11)^2^ | *I think with or without REBOA, these patients have those complications, but if you’re only putting them into severely injured patient REBOA… That is, if you only put the device into each(?) patients, then those patients are more likely to have a shed load of complications, and if they’re not managed well, the patient dies. Participant 6, Clinical Research Fellow, Site 5*  *Yeah, because I’m really good at talking. So the only other issues that I was thinking about just going… so I’ve talked about worrying about side effects haven’t I and that affects your decision making to do it, I think the other thing is that I think operators would be nervous about their first time…Participant 5, Trauma Consultant, Site 6* |
|  | Reputational benefit for the institute associated with being able to recruit patients and deploy REBOA (4)^1 2^ | *I think it’s… the biggest selling point, not to myself and my co-PI colleague, but to the rest of the department was we can’t not be in this trial. We’re going to look silly if we’re not, if we don’t participate in this kind of trial, so there’s some sort of reputational benefit to being part of this. Participant 9, Anaesthetist, Site 8*  *Stuff that would encourage me is that we would be sort of upping our game in trauma by recruiting patients and by contributing to this trial. I think there’s also a bit of a reputational advantage for the department, for the Emergency Department and the trauma service to show other services that, you know, we are taking part in research even during stressful times and I think that’s sort of a badge of honour. Participant 3, Trauma Consultant, Site 6* |
|  | It can be difficult to define exsanguinating haemorrhage (9)^1 2^ | *But I really do think that the randomisation of exsanguinating haemorrhage is libel to clinician interpretation, and clearly it’s been written as such, because it’s a difficult thing to define. Participant 6, Clinical Research Fellow, Site 5*  *Yeah, I think… so this is something that we find exceptionally difficult, and it’s something that I’ve spoken to [CI] about a couple of time and [name] and [name], well just trying to understand who and when should be getting the intervention. So [CI], he’s quite pragmatic when he answers the question, he says, “Look, the trial says they have to have exsanguinating haemorrhage and it be non-compressible” and that’s fine, I get that. Participant 4, Clinical Research Fellow, Site 4* |
|  | I don’t think there are any negative consequences associated with consent processes in REBOA (5)^1^ | *Yes, I’m totally comfortable with it. Participant 6, Clinical Research Fellow, Site 5*  *No, I get out of that bit! That’s the bit that intuitively, personally I would find slightly awkward…“You know that thing we did to you that you didn’t know about -- can I have your consent?” [laughter] Intuitively in my head that feels slightly awkward. Participant 7, Trauma Consultant, Site 3* |
|  | There are both advantages and disadvantages of the broad eligibility criteria (5)^1^ | *I think if it was… one of my points was… the inclusion/exclusion criteria is big and it’s big for a good reason, but it does have some drawbacks to it . Participant 8, Research Nurse, Site 1*  *I think the inclusion criteria is broad and it’s kept broad so that essentially it makes the clinicians position whether to use REBOA or not and that is a problem because you can’t put in words the reasons why clinician would… it will depend on their gut feeling, previous experience, who they have around them, all that kind of stuff, so that can’t be put down on paper, so I think inclusion criteria is pretty broad and it’s kept purposely broad. Participant 3, Trauma Consultant, Site 6* |
| Intentions (13) | I am motivated to take part in REBOA (13)^1 2^ | *Yeah, so I’m all over the trauma stuff, because if it was someone that said my name they would say that I was known for trauma and pre-operative care. Participant 5, Trauma Consultant, Site 6*  *Yeah, but when we heard about it. It wasn’t something I was aware of before we had the…and stuff but yeah, because obviously we’re a major trauma centre so it gained quite a lot of interest around the hospital really, just quite an interesting research trial to be involved in. Participant 1, Research Nurse, Site 6* |
| Optimism (12) | I am hopeful that we can roll out REBOA as a service (8)^2^ | *Again, because it’s something that’s already provided I think that if it became you know, a standard technique then yeah, it would be very easy to extend that. Participant 12, Trauma Surgeon, Site 4*  *In my hospital, I think it would be straightforward because all the trauma surgeons are trained to do an emergency thoracotomy in the resus department to put an aortic clamp on. So, like I mentioned before, this is a different way of putting a clamp on, so I don’t think there would be any resistance from us in terms of performing a REBOA. Some logistics may prevent us getting the REBOA; if the balloon is not available or some technical issues. Then we would just proceed to open the chest and put a clamp in the descending aorta. So, I don’t think in our department it makes a difference. Other departments, other trauma units are not always run by vascular surgeons, so I don’t know how they would be able to proceed with that. That’s my own view. Participant 11, Trauma Surgeon, Site 7* |
|  | I am optimistic we will recruit more patients to the trial (5)^1^ | *I’m really pessimistic. But it goes back to the thought that I had about the epidemiology and the sort of service area for my hospital, so the two hospitals that I’ve been involved in where the REBOA trial is live, one of them is the busiest trauma centre in the country and the other one is [Hospital], my own, and they’re large trauma centres that hoover patients into the trauma centre themselves but that have large geographical expansive areas and if you’re going to bleed to death from the kind of injuries that REBOA is directed at, I think it’s going to happen in the field often before help can get to you, which is a bit of a sad state of affairs, but it is what it is and I’m probably pint half empty on this. Participant 9, Anaesthetist, Site 8*  *Very hopeful and optimistic. So it’s been extended until, I can’t remember when next year but we do have an extension. Participant 8, Research Nurse, Site 1* |
| Behavioural Regulation (12) | We follow checklists, flowcharts and protocols to assist recruitment (7)^1^ | *No, we’ve got a flow sheet which you can follow to work out whether the patient is sick enough and we’ve got a flow sheet that you can follow in order to work out the technicality of how much fluid to put in the various balloons and how far to insert the balloon catheter and I think we’ve taken that straight off the trial website. Participant 9, Anaesthetist, Site 8*  *We have three different documents at STH essentially, one of which is a sort of pre-randomisation checklist: are you thinking of the right things, is this patient appropriate for the trial or not? The second one is a how to actually put a REBOA catheter in, and the third one is a post-randomisation checklist that we have, which is what on earth to do next. Participant 15, Trauma Consultant, Site 8* |
|  | We have a REBOA kit and trolley ready for recruitment (5)^1^ | *Yeah, it was easy. So, we’ve got a trolley, a REBOA trolley in our research room. It is all set up for the procedure. Participant 14, Research Nurse, Site 3*  *We have the kit and we bring that down with us to any of the pre-alerts so we’re ready to deploy it if and when needed. Participant 16, Radiologist, Site 4* |
| Beliefs about Capabilities (9) | There is lots of nervousness around delivering REBOA related to personal abilities (4)^2^ | *I think most people who are in the game are concerned about or have nervousness around is actually once the app says, you know use REBOA, that’s where people’s blood vessels start to go up a bit! In terms of am I going to get it in right? Am I going to do it right, that sort of thing. I think, having never done it in anger before, but only as part of the training scenario. Participant 7, Trauma Consultant, Site 3*    *A lot of my team are a bit apprehensive of it, they feel like if they were to have one they’re like, “Oh, god, try and remember everything”. Participant 8, Research Nurse, Site 1* |
|  | Clinicians have to be confident to deliver REBOA which directly influences recruitment (8)^1 2^ | *Not in the trial, but we have had problems with balloons going up into the vena system or just bad things that you wouldn’t want to happen, probably because people have been trained a bit, but not to a level that they’re comfortable with it. Participant 6, Clinical Research Fellow, Site 5*  *They would have arrived at the trauma call just like, you know, just like the surgeon or the anaesthetist and we could have looked to say this is a potential candidate, they would have instantly pulled up the website and randomised somebody and away it went and that would have taken care of all the regulatory mechanics of the trial. That in itself would still have depended on whether one or both of the consultants were comfortable, were trained in the intervention and/or comfortable in bringing people into the trial, so we’re limited by essentially a number of people that are trained to deliver the randomisation. Participant 9, Anaesthetist, Site 8* |
| Reinforcement (8) | Participating in REBOA is good for personal development (1)^2^ | *I guess everyone gets rewarded in the sense that on some level, I will put that I’ve been a part of this trial in my annual appraisal or whatever and I’ll feel proud about that. That’s a reward in itself. Participant 9, Anaesthetist, Site 8* |
|  | Getting involved in cutting edge research is rewarding (5)^2^ | *Oh I see, okay, so not just to the centre itself. Yeah, so for I think as a trauma centre and even for the experience for the people who are recruiting, for the people who are involved that case, I think they probably feel quite proud that they were able to recruit a patient to a trial during quite stressful conditions and I think that probably improves their confidence in that they are able to conduct research during a stressful time. Participant 3, Trauma Consultant, Site 6*  *What else makes it work well? I think the institution is quite proud of getting REBOA off in the UK, and there are a couple of real enthusiasts. Participant 6, Clinical Research Fellow, Site 5* |
| Goals (8) | We have certain targets and quotas for recruitment (6)^1^ | *These patients are quite rare, so we do our numbers, they tell us we get a patient that is eligible for REBOA every other month, so we do aim to recruit a patient a month, a patient every two months. Participant 14, Research Nurse, Site 3*  *Probably. I don’t know the answer to that. I’m not sure. There probably are, yes. I don’t know, I don’t know what the answer is. I think it’s just that we should trying to… we should be trying to… we should be considering any eligible patient; I think that’s sort of where the standard is. Participant 17, Trauma Consultant, Site 6* |
| Emotion (7) | Stress creates the need for rapid decision-making (6)^1 2^ | *I was just going to describe if we’re doing a chest drain, for example, so popping a tube between the ribs, then we’re used to performing semi-complex procedures under a bit of pressure. I guess that would be the other thing, another barrier is pressure and super sick patients look like they’re about to die, we’ve got to do something, so you probably need to be calm under pressure as well. Participant 15, Trauma Consultant, Site 8*  *So, all we actually did was what we said we would do for all of the code red patients, we would put in a femoral arterial line, regardless, and if they needed randomising, then randomising, and then if they needed REBOA then dilating up from that. I think we got a little bit ahead of ourselves in the heat of the moment and randomised the patient. Participant 7, Trauma Consultant, Site 3* |

*Note*: The domains Social Professional Role and Identity, Knowledge and Intentions were reported frequently, but did not contain content that would suggest that these factors strongly affect recruitment and intervention delivery, nor were conflicting beliefs communicated. The Beliefs about Capabilities domain was regarded as relevant due to the presence of conflicting and strong beliefs around this domain affecting trial processes.

1. REBOA Recruitment

   ^2^ REBOA Intervention delivery [↑](#footnote-ref-1)
